# Recurrent pleuritis with pleural thickening as the manifestation of familial Mediterranean fever

**DOI:** 10.1002/jgf2.331

**Published:** 2020-05-13

**Authors:** Kosuke Ishizuka, Kiyoshi Shikino, Masatomi Ikusaka

**Affiliations:** ^1^ Department of General Medicine Chiba University Hospital Chiba Japan

**Keywords:** respiratory disease, rheumatologic disease

## Abstract

Chest plain computed tomography revealed a high‐density area along the pleura of the right lung base with pleural thickening (arrow heads).

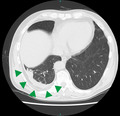

A 67‐year‐old Japanese man presented with recurrent attacks of fever and pleuritic pain for the past 35 years. Symptom resolution occurred within 4‐15 days, and the interval between the attacks ranged from 1 week to 1 year. During the most recent attack, his white blood cell count (11 800/μL) and C‐reactive protein level (19.27 mg/dL) were elevated. Chest plain computed tomography revealed a high‐density area along the pleura of the right lung base with pleural thickening (Figure 1).On the basis of recurrent attacks of high fever and pleuritis, familial Mediterranean fever (FMF) was suspected. There was no evidence of clinical symptoms suggesting amyloidosis in this patient. Administration of colchicine was initiated at a dose of 1.0 mg/d, which resolved the fever and pleuritic pain. In a genetic test, a mutation in exon 10 (M694I) of the Mediterranean fever gene—*MEFV*—was detected, which confirmed the diagnosis of FMF. On the basis of the long febrile period, the patient was classified as having FMF of the atypical type.

**Figure 1:**
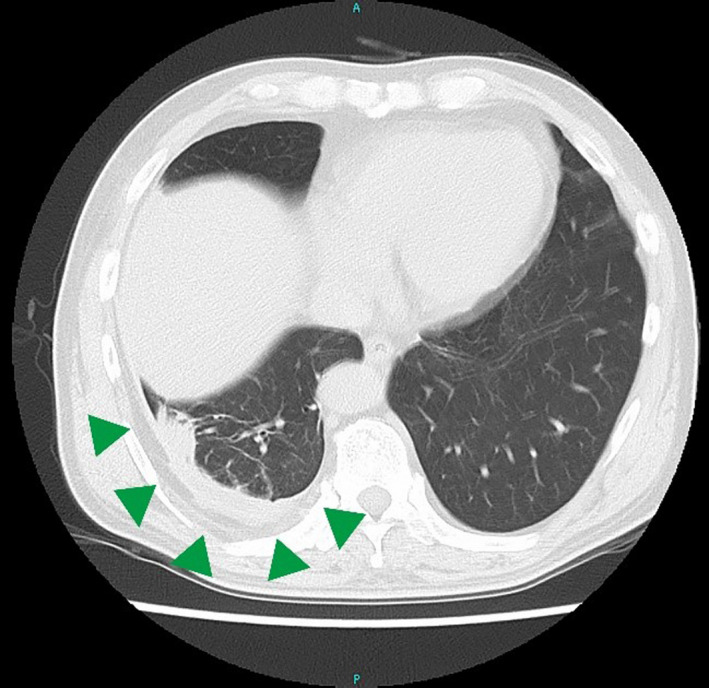
Chest plain computed tomography revealed a high‐density area along the pleura of the right lung base with pleural thickening (arrowheads)

Familial Mediterranean fever is a hereditary autoinflammatory disorder characterized by recurrent attacks of fever and serosal inflammation, including pleuritis. Pleuritis occurs in 38% of all patients with FMF^1^ and typically resolves within 1‐4 days. The prevalence of pleuritis was 23% in patients with FMF of the atypical type,^1^ and patients with exon 10 mutations frequently develop pleuritis.^1^ Chest computed tomography may reveal atelectasis or small pleural effusion, and some patients may also have pleural thickening due to recurrent inflammation.^2^ Thus, FMF must be considered as a differential diagnosis in patients with recurrent pleuritis.

Differential diagnosis of a subsequent pleural thickening occurrence was malignant mesothelioma. Some reports suggested the association of malignant mesothelioma and recurrent FMF.^3^, ^4^ In this case, follow‐up in one‐year chest CT revealed that a high‐density area along the pleura of the right lung base with pleural thickening was not changed at all. This finding did not fit for a subsequent occurrence of malignant mesothelioma.

The reasons for delayed diagnosis from the perspective of diagnostic error illustrate the following two points. The first point is representativeness restraint.^5^ Fever duration of more than 72 hours with atypical symptoms was different from the prototype of familial Mediterranean fever. The second point is Yin‐Yang out.^5^ This patient had been subjected to various tests by previous physicians but had not been diagnosed with any abnormalities leading to FMF diagnosis.

## CONFLICTS OF INTEREST

The authors have stated explicitly that there are no conflicts of interest in connection with this article.

## AUTHOR CONTRIBUTIONS

All authors had access to the data and a role in writing the manuscript.
